# Comparative Analysis of Physicochemical Characteristics, Antioxidant Compound Contents, and Antioxidant Activities of Five Guava (*Psidium guajava* L.) Cultivars Harvested in Korea

**DOI:** 10.3390/foods14213645

**Published:** 2025-10-25

**Authors:** Bohee Choi, Youngjae Shin

**Affiliations:** Department of Food Engineering, Dankook University, Cheonan 31116, Republic of Korea; boandhee@gmail.com

**Keywords:** guava, polyphenol, antioxidants, ascorbic acid, lycopene

## Abstract

Guava (*Psidium guajava* L.) is considered a rich source of bioactive compounds with high antioxidant activity; however, information on cultivars grown in Korea remains limited. This study aimed to compare the physicochemical characteristics, antioxidant compound contents, including lycopene content, and radical scavenging activities of five guava cultivars (‘Gihyun gold no. 2’, ‘Gihyun gold no. 3’, ‘Gihyun green’, ‘Gihyun red’, and ‘Redlee’) cultivated in Eumseong, Korea, during the 2022 season. Significant differences were observed among cultivars in soluble solids, acidity, firmness, and color, as well as in their organic acid, sugar, and polyphenol profiles. Lycopene was detected only in red-fleshed cultivars, with ‘Gihyun gold no. 2’ showing the highest content (5.21 ± 0.20 mg/100 g FW), while ‘Redlee’ exhibited the highest levels of total phenolics (474.92 ± 9.37 mg GAE/100 g FW), ascorbic acid (292.38 ± 4.40 mg/100 g FW), and radical scavenging activities in both assays (432.16 ± 13.37 mg VCE/100 g FW for DPPH and 640.59 ± 50.44 mg VCE/100 g FW for ABTS). In contrast, ‘Gihyun gold no. 2’ consistently showed the lowest antioxidant values. Correlation analysis revealed that total phenolics and ascorbic acid were strongly associated with both DPPH and ABTS radical scavenging activities. These findings indicate that guava cultivars grown in Korea possess high nutritional and functional value, and highlight ‘Redlee’ as a promising source of vitamin C, polyphenols, and lycopene with potential applications in health-promoting foods, nutraceuticals, and value-added product development.

## 1. Introduction

Geographically, tropical fruits originate from equatorial regions in both hemispheres, encompassing tropical and subtropical zones across Asia, Central and South America, Africa, and Oceania. Representative examples include guava, mango, banana, passion fruit, dragon fruit, olive, papaya, atemoya, feijoa, and [1]. These fruits exhibit a high antioxidant potential, primarily due to their abundance of bioactive compounds that exert beneficial effects on human health. In recent years, numerous studies have highlighted the functionality and health-promoting properties of tropical crops [2,3,4,5].

Guava (*Psidium guajava* L.) is a tropical fruit crop that belongs to the Myrtaceae family. It is native to Central America and is currently considered one of the most important commercial fruit crops commonly grown in tropical and subtropical regions. India, Mexico, and Brazil are the leading producers, and the global production of guava was estimated at approximately 40 million tons in 2020 [6]. Guava is available year-round and is a popular fruit for its sweet aroma, taste, and nutritional value [7]. Guava fruit can be consumed fresh, or it can be processed into juices, pastes, jellies, jams, and syrups in the food industry [8]. Guava fruit has a variety of shapes, from spherical to oval, depending on the cultivar. The average diameter is 4–10 cm and the weight is 100–400 g. It has small, hard seeds inside, and the skin color changes from dark green to yellow as the fruit ripens. The flesh color is white, yellow, pink, or red [9].

In Korea, guava is primarily cultivated in Uiryeong, Gyeongsangnam-do; Anseong, Gyeonggi-do; Jeju Island; Eumseong, Chungcheongbuk-do; and Hongseong, Chungcheongnam-do. In this study, five guava cultivars (‘Gihyun gold no. 2’, ‘Gihyun gold no. 3’, ‘Gihyun green’, ‘Gihyun red’, ‘Redlee’) grown in Eumseong County, Chungcheongbuk-do were analyzed. ‘Gihyun gold no. 2’ is oval-shaped resembling a quince, with deep yellow peel and red flesh. ‘Gihyun gold no. 3’ is short oval-shaped, with light yellow-green peel and white flesh. ‘Gihyun green’ is oval-shaped with light yellow peel and white flesh. ‘Gihyun red’ is nearly spherical with peel turning red upon ripening, and white flesh. ‘Redlee’ is short oval-shaped with peel turning red like ‘Gihyun red’ upon complete ripening and has red flesh [10].

Guava is a good source of phenolic compounds, including flavonoids, as well as carotenoids, which contribute to its high antioxidant activity. It also contains dietary fiber, vitamin A, folate, and potassium [11]. Guava is especially known as a good source of ascorbic acid. According to the USDA, guava fruit contains 228.3 mg of ascorbic acid per 100 g, which is about four times the amount found in oranges, which are known to be a good source of ascorbic acid [12]. Carotenoids, a major component of guava, are known to be very potent antioxidants that are efficient in scavenging free radicals. Guava fruit with red flesh contains the fat-soluble antioxidant lycopene. Lycopene is a red pigment found in guava, watermelon, tomato, and pink grapefruit. It is known to have the highest antioxidant activity of all dietary carotenoids [13]. The physiological functions of guava fruit have been reported to include anticancer, antioxidant, anti-bacterial, anti-diabetic, anti-inflammatory, anti-virus, antitumor, and anti-diarrhea [14].

Although research on the functional properties of guava fruit has been reported, research on guava grown in Korea is very rare. Therefore, the objective of this study was to compare and analyze the physicochemical qualities, organic acids, sugars, lycopene, ascorbic acid, and phenolic compound contents, as well as antioxidant activity of five guava cultivars grown in Eumseong, Korea.

## 2. Materials and Methods

### 2.1. Plant Materials

**Figure 1:**
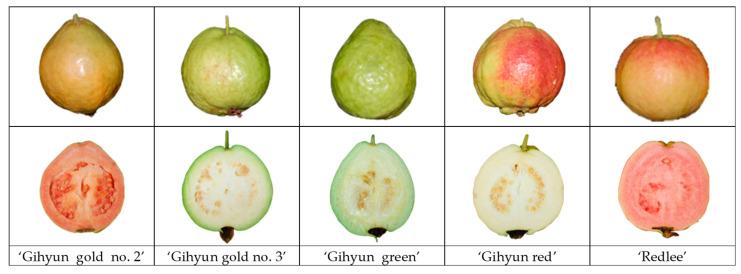
Photograph of the fruit appearance (**top**) and cross-sectional view (**down**) of five guava (*Psidium guajava* L.) cultivars.

The guava fruit used in this study consisted of five cultivars (‘Gihyun gold no. 2’, ‘Gihyun gold no. 3’, ‘Gihyun green’, ‘Gihyun red’, and ‘Redlee’) harvested from the Korean Guava Orchard Farm located in Eumseong, Chungcheongbuk-do. The guava fruit were harvested on 20 October 2022 (Figure 1). For each cultivar, ten fruits of similar marketable maturity were collected from multiple trees within the same orchard, resulting in a total of 50 fruits used for the analyses. This sampling approach ensured appropriate biological replication and minimized variability due to differences in fruit maturity. All fruits were obtained from a single farm and season. The harvested guava fruit was immediately transported to the Food Material Processing Engineering Laboratory at Dankook University. Subsequently, damaged samples were eliminated, and sorted to uniform size. After that, the physicochemical qualities of the guava fruit, such as color, soluble solid content (SSC), titratable acidity, pH, firmness, and lycopene were analyzed. Then, samples were rapidly frozen with liquid nitrogen at −196 °C, and stored in a low temperature freezer at −55 °C. The preserved samples were utilized for the analysis of organic acids, sugars, polyphenols, ascorbic acid, as well as antioxidant compound contents and antioxidant activities.

### 2.2. Color

The color of guava fruit was measured using a colorimeter (Chroma meter CR-400, Minolta, Tokyo, Japan), and expressed as L* (lightness), a* (redness), and b* (yellowness). Color measurements were taken on both the skin (outer peel) and the flesh (inner pulp) of guava fruits. Each sample was subjected to three repeated measurements, and the results were presented as the mean values.

### 2.3. Soluble Solid Content (SSC), Titratable Acidity, pH, and Firmness

To measure SSC, titratable acidity, and pH, whole guava fruits including seeds were homogenized using a commercial blender (GMFC-670, Hanil, Seoul, Republic of Korea) prior to analysis. SSC was determined using a digital refractometer (PAL-1, Atago Co., Ltd., Tokyo, Japan) and expressed in units of °Brix. The titratable acidity was determined by mixing 1 g of homogenized sample with 100 mL of distilled water, adding 3–4 drops of 1% phenolphthalein indicator solution, and titrating with 0.1 N NaOH. The titratable acidity was expressed as a percentage (%) of citric acid equivalent. The pH was measured using a pH meter (Starter 300, Ohaus Co., Ltd., Parsippany, NJ, USA). Firmness was determined using a fruit penetrometer (FHM-1, Demetra Co., Ltd., Tokyo, Japan), and the resistance value at the moment a conical probe (12Φ × 10 mm) penetrated the guava fruit skin was expressed in units of Newtons (N).

### 2.4. Extraction

For sample extraction, 80% acetone was used to extract guava fruit. A portion of the samples was placed in a mortar and ground into a fine powder of uniform particle size by adding an appropriate amount of liquid nitrogen. A 50 g portion of the powdered was mixed with 80% acetone, and homogenization was performed three times for 3 min each time using a commercial blender (JB 3060, Braun, Neu-lsenburg, Germany). The homogenized solution was filtered through Whatman #2 paper filter (Whatman International Ltd., Kent, UK). The filtered solution was concentrated at 45 °C using a rotary evaporator (N-1300, Eyela, Tokyo, Japan). The concentrated extract was preserved in a low temperature freezer at −55 °C and utilized for the analysis of chemical compositions, total flavonoid content, total phenolic content, individual phenolic compounds, and radical scavenging activity assays.

### 2.5. Quantification of Individual Organic Acids

The organic acid compositions of guava fruit were analyzed using the method of Kim and Shin [15] with some modifications. The sample extracts were diluted with distilled water at appropriate ratios and the filtered liquid, passed through a 0.45 µm syringe filter, was used for analysis. HPLC (Agilent 1200 series, Agilent Technol., Wilmington, DE, USA), with auto sampler, quaternary pump, DAD was utilized. The separation of individual organic acids was conducted at 25 °C using a Prevail organic acid column (250 × 4.6 mm, 5 µm, Hichrom Ltd., Reading, UK). A mobile phase of 25 mM KH_2_PO_4_ (pH 2.5) was used, with a flow rate set at 1 mL/min. The sample injection volume was 10 µL. Detection was performed at a wavelength of 210 nm using a diode array detector (DAD). Oxalic acid, tartaric acid, malic acid, lactic acid, acetic acid, citric acid, succinic acid, and fumaric acid were used for standard calibration curves. The organic acid contents of guava fruit were expressed in units of mg/100 g fresh weight (FW).

### 2.6. Quantification of Individual Sugars

The sugar composition of guava fruit was analyzed according to the method by Kim and Shin [15]. Sample extracts were diluted with distilled water and the filtered liquid, passed through a 0.45 µm syringe filter, was used for analysis. HPLC (UltiMate 3000, Thermo Fisher Scientific, Waltham, MA, USA), with a RefractoMax 520 refractive index (RI) detector (ERC Inc., Saitama, Japan) was utilized. The separation of individual sugar was conducted at 30 °C using a Asahipak NH2P-50 4E column (250 × 4.6 mm, 5 µm, Waters Corp., Milford, MA, USA). A mobile phase of 75% acetonitrile in distilled water was used, with a flow rate set at 1 mL/min, and the sample injection volume was 10 µL. Standard calibration curves used fructose, glucose, and sucrose, and the sugar contents in guava fruit were expressed in units of g/100 g FW.

### 2.7. Total Flavonoid Analysis

Total flavonoid contents of guava fruit were measured by a colorimetric assay [16]. A properly diluted sample extract of 1 mL was mixed with 4 mL of deionized water. Then 0.3 mL of 5% NaNO_2_ was added to the mixture and allowed to react at room temperature for 5 min. After that, 0.3 mL of 10% AlCl_3_ was added and mixed, and reacted at room temperature for 6 min. Finally, 2 mL of 1N NaOH and 2.4 mL of deionized water were added to adjust the total volume to 10 mL. The test solution was measured for absorbance at 510 nm using a spectrophotometer (Optizen POP, Mecasys, Daejeon, Republic of Korea). The total flavonoid contents of guava fruit were expressed as mg catechin equivalents (CE)/100 g FW.

### 2.8. Total Phenolic Analysis

Total phenolic contents of guava fruit were measured using a modified Folin–Ciocalteu colorimetric method [17]. A properly diluted sample extract of 0.2 mL was mixed with 2.6 mL of deionized water. Then 0.2 mL of Folin–Ciocalteu’s phenol reagent was added to the mixture and allowed to react at room temperature for 6 min. Afterward, 2 mL of 7% NaCO_3_ was added to the mixture to adjust the total volume to 5 mL. Finally, after reacting at room temperature for 90 min, the test solution was measured for absorbance at 750 nm using a spectrophotometer. The phenolic contents of guava fruit were expressed as mg gallic acid equivalents (GAE)/100 g FW.

### 2.9. Polyphenol Analysis

Individual polyphenol of guava fruit was analyzed using a modified method by Yang et al. [17]. The sample extracts were diluted using a dilution solution (KH_2_PO_4_:methanol:water = 2:3:15) and filtered through a 0.45 µm syringe filter for analysis. HPLC (Agilent 1200 series, Agilent Technol., Wilmington, DE, USA), with auto sampler, quaternary pump, DAD was used. The separation of individual polyphenol was conducted at 40 °C using a Zorbax Eclipse XDB C18 column (150 × 4.6 mm, 5 µm, Agilent Technol., Wilmington, DC, USA). As the mobile phase, 3% acetic acid (solvent A) and 100% methanol (solvent B) were used, and the solvent gradient was applied as follows: 100% A/0% B at 0 min, 90% A/10% B at 4 min, 45% A/55% B at 15 min, 100% A/0% B at 18 min. The flow rate was set to 0.9 mL/min, and the injection volume was 10 µL. Detection was performed at a wavelength of 280 nm using a diode array detector (DAD). Standard calibration curves used gallic acid, chlorogenic acid, (+)-catechin hydrate, epigallocatechin gallate, epicatechin, ferulic acid, rutin hydrate, rosmarinic acid, and quercetin. Polyphenol contents of guava fruit were expressed as mg/100 g FW.

### 2.10. Lycopene Analysis

The lycopene content of guava fruit was analyzed using modified traditional lycopene quantification methods [18,19,20,21]. A small amount of deionized water, methanol, and celite powder (No. 545) were added to guava fruit, and the mixture was stirred. A 100 mL volumetric flask was equipped with a glass filter (3G3) and pressure was applied using an aspirator (AAA71015, Jeio Tech Co., Ltd., Daejeon, Republic of Korea). Then the guava mixture was poured into the glass filter. Concurrently, methanol was added, and decompression filtered until the extraction solvent changed from yellow to colorless. The glass filter was connected to a 100 mL volumetric flask, and benzene was added until the red color of the residual extract turned transparent. The final solution volume was adjusted to 100 mL by adding benzene. The test solution was measured for absorbance at 487 nm using a spectrophotometer. Lycopene contents of guava fruit were expressed as mg/100 g FW.

$$\mathrm{Lycopene}\;(\mathrm{mg}/100\;g)=\frac{\left(C\times D\times 1000\right)}{S\times 1000}$$

C = Concentration of lycopene (µg/mL), estimated from the calibration curve.

D = Dilution factor.

S = Amount of collected samples (g).

### 2.11. Total Ascorbic Acid Analysis

The total ascorbic acid contents of guava fruit were analyzed using the DNPH (dinitrophenylhydrazine) method of Terada et al. [22]. Frozen guava fruit samples were ground into a fine powder using liquid nitrogen, and 5 g of the powdered sample was mixed with 100 mL of 6% metaphosphoric acid (buffer). Then, centrifugation was performed at 15,000 rpm for 20 min, and only the supernatant was used as the test solution. One milliliter of test solution was mixed with 0.05 mL of 2% DCIP (2,6-dichlorophenolindophenol), and the mixture was reacted for one hour at room temperature. Subsequently, 1 mL of 2% thiourea and 0.5 mL of 2% DNPH were added to the mixture, and reacted for three hours in an incubator at 60 °C. After the reaction, the test tube containing the mixture was cooled down by placing it in ice, and 2.5 mL of 90% H_2_SO_4_ was added. H_2_SO_4_ was slowly added to remove osazone. The test solution was measured for absorbance at 540 nm using a spectrophotometer. The total ascorbic acid content of guava fruit was expressed as mg/100 g FW.

### 2.12. DPPH Radical Scavenging Activity Analysis

The DPPH radical scavenging activity of guava fruit was measured using a modified method by Yang et al. [17]. A properly diluted sample extract of 50 µL was mixed with 2950 µL of 0.2 mM DPPH solution, and the mixture was reacted at room temperature for 30 min. After the reaction, absorbance was measured at a wavelength of 517 nm using a spectrophotometer. The DPPH radical scavenging activity of guava fruit was expressed as mg vitamin C equivalents (VCE)/100 g FW.

### 2.13. ABTS Radical Scavenging Activity Analysis

The ABTS radical scavenging activity of guava fruits was measured by the method described in Hwang et al. [23]. A mixture of 1 mM 2,2′-Azobis (2-amidinopropane) dihydrochloride (AAPH), 1X phosphate-buffer saline (PBS) solution, and 2.5 mM ABTS was prepared, and this mixture was reacted in a 70 °C water bath for 40 min to create the ABTS solution. A properly diluted sample extract of 20 µL was mixed with 980 µL of the ABTS solution and reacted for 10 min in a 37 °C incubator. After the reaction, absorbance was measured at a wavelength of 734 nm using a spectrophotometer. The ABTS radical scavenging activity of guava fruit was expressed as mg VCE/100 g FW.

### 2.14. Statistical Analysis

Statistical analysis of each experiment was performed by analysis of variance (ANOVA) using the SPSS 27 program (SPSS Inc., Chicago, IL, USA), and significant differences in the results were expressed by Duncan’s multiple range test (*p* < 0.05). The correlation of the mean values for each parameter was analyzed using Pearson’s correlation coefficient, and the data were expressed as the mean ± standard deviation of the values from triplicate determination.

## 3. Results and Discussion

### 3.1. Physicochemical Qualities, Organic Acids, and Sugars of Guava Fruit

#### 3.1.1. Color

The color of guava fruit was measured separately on skin and flesh parts, and the results are shown in Table 1. In guava skin, the a* value was significantly highest in ‘Gihyun red’ (4.59 ± 4.87), which has a relatively red skin color. ‘Gihyun gold no. 3’ (−16.30 ± 0.70) and ‘Gihyun green’ (−16.23 ± 1.46), which are close to light green in skin color, were significantly the lowest. The b* value was highest in ‘Gihyun gold no. 2’ (40.73 ± 1.21), which has a deeper yellow in skin color compared to other cultivars. In guava flesh, the L* value was significantly higher in white-flesh cultivars such as ‘Gihyun gold no. 3’ (83.06 ± 1.70), ‘Gihyun red’ (82.35 ± 0.97), and ‘Gihyun green’ (82.32 ± 2.40) compared to red-flesh cultivars. The a* value of guava flesh was higher in red-flesh cultivars, such as ‘Redlee’ (25.82 ± 1.66) and ‘Gihyun gold no. 2’ (21.75 ± 1.23). Previous studies have shown that guava color varies markedly with maturity, as chlorophyll degradation and carotenoid biosynthesis occur during ripening [24]. Thus, the color differences among cultivars observed in this study primarily reflect genetic variation in pigment biosynthesis. All fruits were harvested at the same marketable maturity suitable for fresh consumption, thereby minimizing ripeness-related effects on color. Therefore, the observed variation in skin and flesh color is an inherent cultivar trait rather than a reflection of under- or over-ripeness. Since color is one of the most important visual quality traits influencing consumer preference, these findings highlight the role of cultivar selection in determining guava appearance and market value [24,25].

#### 3.1.2. Soluble Solid Content (SSC), Titratable Acidity, pH, and Firmness

The SSC, titratable acidity, pH, and firmness of guava fruit are shown in Table 2. The SSC of guava fruit ranged from 10.50 to 13.90 °Brix. ‘Gihyun red’ was the significantly highest (13.90 ± 0.00 °Brix), followed by ‘Redlee’ > ‘Gihyun gold no. 2’ > ‘Gihyun gold no. 3’ > ‘Gihyun green’. According to Kumari et al. [26], SSC of commercially harvested guavas in India ranged from 9.98 to 13.10 °Brix. In another study, the SSC of 15 different guava varieties varied in the range of 9.3 to 13.7 °Brix, depending on their genetic types.

The titratable acidity of guava fruit was 1.33–1.76%. ‘Gihyun gold no. 3’ was the significantly highest (1.76 ± 0.04%), while ‘Gihyun green’ was the significantly lowest (1.33 ± 0.01%). The pH of guava fruit was below pH 4 in all cultivars. ‘Redlee’ (3.99 ± 0.01) and ‘Gihyun gold no. 2’ (3.98 ± 0.01) were the significantly highest.

According to the study by Moon et al. [27] on twenty-seven guava accessions at different developmental stages, the variation in pulp pH (3.0–4.6) was not associated with fruit maturity, whereas titratable acidity (0.17–3.6%) tended to decline as the fruit ripened. In addition to fruit maturity, these physicochemical properties are not only determined by cultivar but are also influenced by multiple factors. Environmental conditions such as climate, soil composition, and water availability can markedly affect fruit acidity, sugar accumulation, and pigment biosynthesis [27,28,29].

The firmness of guava fruits in this study ranged from 6.83 to 9.28 N, with ‘Gihyun gold no. 3’ exhibiting the highest value (9.28 ± 0.11 N) and ‘Gihyun green’ the lowest (6.83 ± 0.61 N). Firmness is known to be closely related to pectin composition. Because guava is a climacteric fruit, tissue softening occurs during ripening due to the enzymatic degradation of cell wall polysaccharides. In particular, the conversion of insoluble protopectin into water-soluble pectin and pectinic acid has been identified as a major factor contributing to the loss of firmness [30,31].

#### 3.1.3. Organic Acid Content

The organic acid compositions of guava fruit are shown in Table 3. In this study, seven organic acid standards (oxalic acid, tartaric acid, malic acid, acetic acid, citric acid, succinic acid and fumaric acid) were used. However, tartaric acid and succinic acid were not detected in all cultivars. These organic acids contribute significantly to the sourness and tartness that balance the sweetness from sugars, creating a harmonious flavor profile that is key to consumer preference and overall fruit palatability.

The total organic acid content of guava fruit was highest in ‘Gihyun gold no. 3’ (674.67 mg/100 g FW), and lowest in ‘Gihyun green’ (481.31 mg/100 g FW). Citric acid and malic acid were the primary organic acids in guava fruit. Citric acid content was significantly highest in ‘Gihyun gold no. 3’ (620.87 ± 2.45 mg/100 g FW). Malic acid was significantly highest in ‘Gihyun gold no. 2’ (178.56 ± 0.16 mg/100 g FW), followed by ‘Redlee’ > ‘Gihyun red’ > ‘Gihyun green’ > Gihyun gold no. 3’. Oxalic acid showed relatively lower content compared to lactic acid, citric acid, and malic acid in all cultivars. Acetic acid and fumaric acid were either not detected or present only in trace amounts, and their occurrence appeared to be cultivar-dependent. Citric and malic acids are common organic acids in most fruits, although the predominant acids vary by species. In guava, Lee et al. [32] identified citric and malic acids as the dominant acids, while Muñoz-Arrieta et al. [33] reported citric, succinic, malic, and tartaric acids, with citric acid being the most abundant. Similarly, Rojas-Garbanzo et al. [34] found that citric acid accounted for nearly 50% of the total organic acid content in guava. In addition, during fruit ripening, the decrease in organic acid content often correlates with an increase in sugar content, which naturally enhances sweetness and reduces sourness, modifying consumer perception and eating quality. Understanding these dynamics is important for postharvest handling and processing of guava to optimize flavor and nutritional quality [35].

#### 3.1.4. Sugar Content

The sugar profiles of guava fruit are shown in Table 4. In this study, three sugar standards (fructose, glucose, and sucrose) were used. Sugar composition plays a crucial role in determining the overall flavor balance and consumer acceptability of fresh fruits [36]. The ratio of glucose, fructose, and sucrose not only influences perceived sweetness but also contributes to the characteristic aroma and mouthfeel of guava. Among the guava cultivars, ‘Gihyun red’ had the highest total sugar content, followed by ‘Redlee’, ‘Gihyun gold no. 2’, ‘Gihyun green’, and ‘Gihyun gold no. 3’. Fructose was significantly the highest in ‘Gihyun red’ (5.41 ± 0.16 g/100 g FW) and glucose was significantly higher in ‘Gihyun gold no. 2’ (3.48 ± 0.03%) and ‘Gihyun red’ (3.44 ± 0.10 g/100 g FW). ‘Redlee’ had significantly higher sucrose content (4.84 ± 0.05 g/100 g FW) compared to other cultivars, and this higher sucrose level is generally associated with enhanced sweetness perception and consumer preference, especially when balanced by moderate acidity that provides a pleasant flavor contrast. Fructose, glucose, and sucrose were detected in all cultivars, but the dominant sugars varied among cultivars. In ‘Redlee’, sucrose was the main sugar, accounting for 56.15% of the total sugar content, while in other cultivars, fructose was the predominant sugar.

Many studies have confirmed that fructose, glucose, and sucrose are the main sugars commonly found in fruits. The sugar profile of fruits varies due to factors such as temperature, solar radiation, harvest, storage conditions, genetic factors, and more [37]. The average sugar content of fructose and glucose in guava harvested from four different regions in Kenya (Rift Valley, Western, Coast, Eastern) was 2.81 and 1.11 g/100 g FW, respectively, with variations in sugar content depending on the region [38]. In the study by Sviech et al. [39], the glucose, sucrose, and fructose content in fresh guava pulp were 246.4, 139.9, and 20.4 mg/g FW, respectively, and glucose was the main sugar. Our study also showed differences in individual sugar composition and content among cultivars, and the total sugar content of ‘Gihyun red’ was the highest. This pattern is consistent with the soluble solid content results.

### 3.2. Antioxidant Compound Contents and Antioxidant Activity Analysis of Guava Fruit

#### 3.2.1. Total Flavonoid and Phenolic Contents

**Figure 2:**
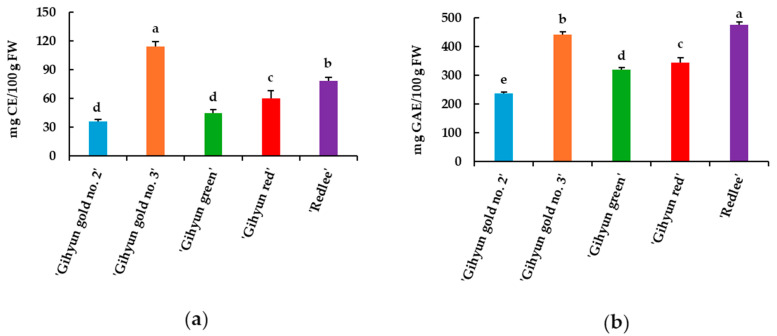
Total flavonoid contents (**a**) and total phenolic contents (**b**) of guava (*Psidium guajava* L.) fruit. Values represent the mean ± SD of triplicate technical measurements obtained from ten biological fruits. Different letters are significant differences by Duncan’s multiple range test (*p* < 0.05).

The total flavonoid contents of guava fruit are shown in Figure 2a. ‘Gihyun gold no. 3’ had significantly higher content (114.40 ± 4.98 mg CE/100 g FW) compared to the other cultivars. ‘Redlee’ and ‘Gihyun red’ and had contents of 78.65 ± 3.56 and 60.35 ± 8.14 mg CE/100 g FW, respectively. ‘Gihyun green’ (44.84 ± 3.74 mg CE/100 g FW) and ‘Gihyun gold no. 2’ (35.92 ± 2.58 mg CE/100 g FW) had relatively lower contents.

According to Patel et al. [40], the total flavonoid content of fresh guava extract was 202.01 mg CE/100 g FW, which was higher content than in our study. In contrast, Chauhan et al. [41] reported a lower total flavonoid content of 16.4 to 19.4 mg/100 g FW compared to our study. According to Sanguansil et al. [42], the total flavonoid content of 24 guava varieties ranged from a minimum of 24 mg CE/100 g FW to a maximum of 64.9 mg CE/100 g FW.

The total phenolic contents of guava fruits are shown in Figure 2b. ‘Redlee’ exhibited the highest value (474.92 ± 9.37 mg GAE/100 g FW), followed by ‘Gihyun gold no. 3’, ‘Gihyun red’, and ‘Gihyun green’, while ‘Gihyun gold no. 2’ showed the lowest content. These results indicate that total phenolic content varied markedly among guava cultivars. In the study by Omayio et al. [43], the total phenolic content of red-fleshed guava and white-fleshed guava was 1649.14 and 1386.54 mg GAE/100 g DW, respectively. Red-fleshed guava had higher phenolic levels compared to white-fleshed guava. However, in our study, the difference in total phenolic content of guava was not related to the color of the fruit flesh. According to Yousaf et al. [36], the total phenolic contents of eight guava varieties ranged from 94.06 to 190.64 mg GAE/100 g DW. These contents were lower compared to our study. Lim et al. [44] found that guava’s total phenolic content varies depending on its ripeness. This phenomenon can be attributed to the fact that during the early stages of fruit development, the phenolic content increases due to biosynthetic processes, but in the later stages, it can decrease due to the increased activity of polyphenol oxidase. Therefore, our results suggest that variations in total phenolic content may be influenced by factors such as cultivar differences, ripening stage, and polyphenol oxidase activity.

#### 3.2.2. Polyphenol Content

The polyphenol contents of guava fruit are shown in Table 5. Polyphenols are major phytochemicals that contribute to the antioxidant activity as secondary metabolites in various plant species [45]. The polyphenols in guava fruit were identified as (+)-catechin hydrate, rutin hydrate, epigallocatechin gallate, ferulic acid, gallic acid, epicatechin, and rosmarinic acid. The main polyphenol in ‘Gihyun gold no. 3’, ‘Gihyun green’, and ‘Gihyun red’ was (+)-catechin hydrate, while ‘Redlee’ ‘and Gihyun gold no. 2’ were rutin hydrate.

(+)-Catechin hydrate was significantly highest in ‘Gihyun gold no. 3’ (55.55 ± 0.53 mg/100 g FW), and rutin hydrate had the highest content in ‘Redlee’ (64.81 ± 0.73 mg/100 g FW). Epigallocatechin gallate, ferulic acid and gallic acid were also detected in all cultivars. Interestingly, epicatechin and rosmarinic acid were only detected in ‘Gihyun gold no. 2’ and ‘Gihyun gold no. 3’, and chlorogenic acid and quercetin were not detected in all cultivars. According to Fu et al. [46], guava polyphenols, including (+)-catechin, quercetin, gallic acid, (-)-epicatechin, luteolin, and kaempferol, were identified, with (+)-catechin showing the highest content among them. In another study, flavonoids such as rutin, catechin, kaempferol, and quercetin were identified in guava, with rutin reported to have the highest content [47,48]. These studies support our results by indicating that the main polyphenols of guava are (+)-catechin hydrate and rutin hydrate.

In our study, ‘Redlee’ showed the highest total polyphenol content among guava cultivars, followed by ‘Gihyun gold no. 3’, ‘Gihyun red’, ‘Gihyun gold no. 2’, and ‘Gihyun green’. The total polyphenol contents exhibited a similar trend to the total phenolic contents. Additionally, the individual polyphenol composition and content of guava fruit varied depending on the cultivars. Overall, all cultivars had higher contents of (+)-catechin hydrate and rutin hydrate, and these polyphenol compounds were considered to significantly contribute to the total phenolic contents. In addition, environmental conditions exert a significant influence on the metabolite profiles of guava cultivars, shaping their physicochemical characteristics and functional properties. Climatic factors such as temperature, light intensity, and duration, as well as agronomic practices including irrigation and soil management, interact with genetic determinants to regulate metabolite biosynthesis, accumulation, and degradation [49].

#### 3.2.3. Lycopene Content

The lycopene contents of guava fruits are shown in Table 6. Lycopene was detected only in the red-fleshed cultivars, with ‘Gihyun gold no. 2’ (5.21 ± 0.20 mg/100 g FW) containing more than ‘Redlee’ (3.60 ± 0.77 mg/100 g FW). The absence of lycopene in white-fleshed cultivars indicates that this carotenoid is closely associated with red pigmentation, consistent with its established role as a major pigment in red-colored fruits such as tomato and watermelon. In red-fleshed guava, lycopene is also the principal pigment, accounting for approximately 86% of the total carotenoid content [50]. According to Musaa et al. [51], the lycopene contents of the pink guavas (‘Sungkai’ and ‘Semenyih’) were reported to be 4.05 and 5.28 mg/100 g FW, respectively, which are comparable to the levels observed in the red-fleshed guava cultivars in our study. Kumari et al. [26] found that lycopene was only detected in the ‘Lalit’ cultivar (17.69 μg/100 g FW), while it was not detected in the other cultivars. These findings align with our study, which observed variations in lycopene content among different cultivars. Both tomato and guava are well-known as dietary sources of lycopene. According to Nwaichi et al. and Amorim et al. [52,53], guava had a relatively higher lycopene content compared to tomatoes. Therefore, the red-fleshed guava cultivars (‘Gihyun gold no. 2’ and ‘Redlee’) are considered to be excellent sources of lycopene.

The use of benzene as an extraction solvent has been widely applied in traditional lycopene quantification protocols due to its high extraction efficiency; however, its toxicity and environmental impact have raised increasing concerns. Recent research trends emphasize green extraction approaches that promote the use of alternative, bio-based solvents to replace toxic and hazardous chemicals such as benzene or chlorinated solvents [54,55,56,57].

#### 3.2.4. Total Ascorbic Acid Content

The total ascorbic acid contents of guava fruits are presented in Table 6. ‘Redlee’ showed the highest level (292.38 ± 4.40 mg/100 g FW), followed by ‘Gihyun green’ (214.50 ± 6.10 mg/100 g FW), ‘Gihyun gold no. 3’ (198.27 ± 3.91 mg/100 g FW), and ‘Gihyun red’ (164.96 ± 4.27 mg/100 g FW). The ascorbic acid content varied considerably among cultivars, with more than a twofold difference observed between ‘Redlee’ and ‘Gihyun gold no. 2’. In the study by Jawaheer et al. [58], the average ascorbic acid contents of the ‘Labourdonnais White’ and ‘Hawaiian’ guava varieties were 201.1 and 95.4 mg/100 g FW, respectively. Thaipong et al. [59] also reported a wide range (174.2–396.7 mg/100 g FW) across cultivars, confirming the cultivar-dependent variability, consistent with our results. Compared with other tropical fruits, guava has been reported to contain relatively high ascorbic acid levels than banana, mangosteen, mango, and pineapple [60,61]. In our study, all guava cultivars exhibited higher ascorbic acid contents than those previously reported, confirming guava as an excellent dietary source of vitamin C.

The ascorbic acid content of fruits is influenced by various factors, including species, variety, horticultural practices, sun exposure, photosynthetic processes, and environmental temperature. Moreover, ascorbic acid is highly sensitive to oxidative degradation, and its retention may be further affected by enzymatic activity, postharvest handling conditions, and cultivar differences [62].

#### 3.2.5. DPPH and ABTS Radical Scavenging Activity

**Figure 3:**
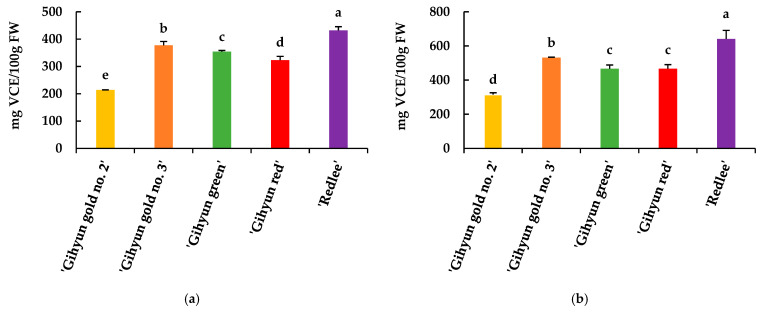
DPPH radical scavenging activities (**a**) and ABTS radical scavenging activities (**b**) of guava (*Psidium guajava* L.) fruit. Values represent the mean ± SD of triplicate technical measurements obtained from ten biological fruits. Different letters are significant differences by Duncan’s multiple range test (*p* < 0.05).

The antioxidant activities of guava fruits, measured by DPPH and ABTS radical scavenging assays, are shown in Figure 3. Among the cultivars, ‘Redlee’ exhibited the highest activities in both assays (432.16 ± 13.37 mg VCE/100 g FW for DPPH and 640.59 ± 50.44 mg VCE/100 g FW for ABTS). ‘Gihyun gold no. 3’ showed the second highest activities (377.26 ± 14.07 and 531.11 ± 3.39 mg VCE/100 g FW, respectively), followed by ‘Gihyun green’ (354.11 ± 4.59 and 466.52 ± 22.35 mg VCE/100 g FW) and ‘Gihyun red’ (322.63 ± 14.33 and 466.21 ± 24.49 mg VCE/100 g FW), which had similar values. In contrast, ‘Gihyun gold no. 2’ displayed the lowest activities in both assays (213.99 ± 0.38 and 315.33 ± 10.52 mg VCE/100 g FW). Overall, significant differences were observed among the cultivars, and the ranking of antioxidant activity was consistent across both DPPH and ABTS assays.

Samee et al. and Du et al. [63,64] reported a significant correlation among total phenolic content, ascorbic acid content, and antioxidant activity. Similarly, Suwanwong et al. [65] also reported a significant relationship between total flavonoid content, total phenolic content, and ascorbic acid content and antioxidant capacity in guava extracts. In our study, ‘Redlee’ had relatively higher levels of organic acid, total phenolics, and total ascorbic acid content, and lycopene content compared to other cultivars. Therefore, it can be inferred that antioxidant compounds significantly influenced the DPPH radical scavenging activity of ‘Redlee’. Alothman et al. [66] established a positive correlation between the total phenolics content and antioxidant capacity in guava extracts. They observed an increase in DPPH radical inhibition with higher total phenolics, consistent with our results.

According to Floegel et al. [67], ABTS radical scavenging activity demonstrated greater antioxidant capacity compared to DPPH radical scavenging activity. Additionally, a strong positive correlation was reported between the two methods. These findings support our study, where ABTS radical scavenging activity was measured higher than DPPH radical scavenging activity. Similar to DPPH radical scavenging activity, ‘Redlee’, which had the highest amount of antioxidant compounds, exhibited the highest scavenging activity in ABTS radical scavenging activity as well. Therefore, ABTS radical scavenging activity can be attributed to the antioxidant compounds, including diverse polyphenols, and total ascorbic acid content found in guava fruit. In addition, agronomic factors such as fertilization, water availability, and harvest timing also impact metabolite profiles by influencing stress responses and metabolic fluxes within fruit and vegetables. For instance, controlled water stress can enhance secondary metabolite production as part of the plant’s adaptive mechanisms, improving fruit quality traits like flavor and antioxidant activity [49]. Understanding these environmental effects is essential for optimizing cultivation practices aimed at enhancing the desired biochemical attributes in guava fruits.

### 3.3. Pearson’s Correlation

**Figure 4:**
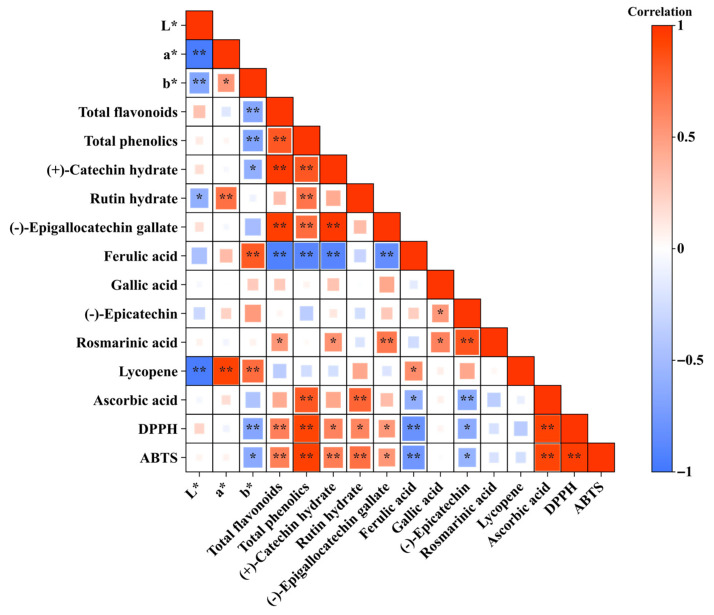
Pearson’s correlation heatmap showing relationships among color parameters, antioxidant compounds, and antioxidant activities of guava fruits. The scale represents the Pearson’s correlation coefficient (*R*), where blue indicates negative correlations and red indicates positive correlations, with color intensity corresponding to correlation strength. Asterisks denote statistical significance (* *p* < 0.05, ** *p* < 0.01). The results were annotated by the online tools of Chiplot.

The correlations among physicochemical quality, organic acids, sugars, antioxidant compounds, and activities of guava fruit are shown in Figure 4. The titratable acidity of guava fruit showed a very high correlation with citric acid (*R* = 0.909) among various organic acids. This indicates that the sour taste of guava fruit is significantly influenced by its citric acid content. The major phytochemical in guava fruit, lycopene, exhibited a strong positive correlation with the a* value (*R* = 0.935). This suggests a significant relationship between lycopene and the redness values, as lycopene is the component responsible for the red color in guava.

The total flavonoids of guava fruit showed a strong positive correlation with (+)-catechin hydrate (*R* = 0.978) and epigallocatechin gallate (*R* = 0.955). It also exhibited a high correlation with total phenolics (*R* = 0.834). Therefore, it is inferred that the majority of phenolics present in guava fruit are flavonoids, with (+)-catechin hydrate comprising the largest portion among them. Both total phenolics and total ascorbic acid of guava fruit showed strong positive correlations with radical scavenging activities. The total phenolics were highly correlated with DPPH (R = 0.925) and ABTS (R = 0.945) radical scavenging activities, while total ascorbic acid also exhibited strong correlations with DPPH (R = 0.935) and ABTS (R = 0.919) radical scavenging activities.

## 4. Conclusions

The present study revealed substantial variability in physicochemical characteristics chemical compositions, and antioxidant potential among guava cultivars grown in Eumseong, Korea, during the 2022 season. The red-fleshed cultivar ‘Redlee’ exhibited the highest levels of ascorbic acid, polyphenols, and lycopene, indicating its potential as a valuable functional food resource. These findings not only provide fundamental data for the selection and utilization of guava cultivars in Korea but also support their broader potential in nutrition and health promotion. However, as this study was conducted using fruits harvested from a single location and season, and did not assess postharvest stability or bioavailability of the bioactive compounds, further studies are needed to confirm these findings under broader cultivation and storage conditions.

## Figures and Tables

**Table 1:** Hunter L*, a*, and b* values of guava (*Psidium guajava* L.) skin and flesh.

Cultivar	Part	L* (Lightness)	a* (Redness)	b* (Yellowness)
‘Gihyun gold no. 2’	Skin	68.28 ± 1.36 ^b^	−2.94 ± 2.89 ^d^	40.73 ± 1.21 ^a^
	Flesh	63.40 ± 1.30 ^bc^	21.75 ± 1.23 ^b^	30.07 ± 1.69 ^d^
‘Gihyun gold no. 3’	Skin	60.31 ± 0.64 ^c^	−16.30 ± 0.70 ^e^	35.36 ± 0.47 ^c^
	Flesh	83.06 ± 1.70 ^a^	−0.37 ± 0.41 ^d^	14.27 ± 2.32 ^f^
‘Gihyun green’	Skin	66.64 ± 5.80 ^b^	−16.23 ± 1.46 ^e^	38.26 ± 3.08 ^abc^
	Flesh	82.32 ± 2.40 ^a^	−2.62 ± 1.38 ^d^	20.44 ± 3.84 ^e^
‘Gihyun red’	Skin	65.83 ± 5.12 ^b^	4.59 ± 4.87 ^c^	36.05 ± 3.17 ^bc^
	Flesh	82.35 ± 0.97 ^a^	−0.13 ± 0.34 ^d^	14.50 ± 2.41 ^f^
‘Redlee’	Skin	67.95 ± 0.52 ^b^	−2.91 ± 3.34 ^d^	40.05 ± 1.15 ^ab^
	Flesh	64.36 ± 2.63 ^bc^	25.82 ± 1.66 ^a^	18.42 ± 0.85 ^e^

Results are the mean values ± standard deviation (SD) from three technical measurements of ten biological fruits; means in the same column with superscript with different letters (a, b, c, d, e and f) are significantly different at *p* < 0.05.

**Table 2:** Soluble solid content (SSC), titratable acidity, pH, and firmness of guava (*Psidium guajava* L.) fruit.

Cultivar	SSC (°Brix)	Titratable Acidity (%)	pH	Firmness (N)
‘Gihyun gold no. 2’	11.77 ± 0.12 ^c^	1.57 ± 0.02 ^b^	3.98 ± 0.01 ^a^	7.65 ± 0.34 ^c^
‘Gihyun gold no. 3’	10.67 ± 0.12 ^d^	1.76 ± 0.04 ^a^	3.82 ± 0.01 ^c^	9.28 ± 0.11 ^a^
‘Gihyun green’	10.50 ± 0.17 ^d^	1.33 ± 0.01 ^d^	3.89 ± 0.02 ^b^	6.83 ± 0.61 ^d^
‘Gihyun red’	13.90 ± 0.00 ^a^	1.54 ± 0.03 ^b^	3.77 ± 0.01 ^d^	8.50 ± 0.58 ^ab^
‘Redlee’	12.70 ± 0.10 ^b^	1.39 ± 0.02 ^c^	3.99 ± 0.01 ^a^	8.39 ± 0.33 ^bc^

Results are the mean values ± SD from three technical measurements of ten biological fruits; means in the same column with superscript with different letters (a, b, c, and d) are significantly different at *p* < 0.05.

**Table 3:** Individual organic acid contents (mg/100 g FW) of guava (*Psidium guajava* L.) fruit.

Cultivar	Citric Acid	Malic Acid	Oxalic Acid	Acetic Acid	Fumaric Acid	Total Sum
‘Gihyun gold no. 2’	439.08 ± 0.90 ^b^	178.56 ± 0.16 ^a^	13.20 ± 0.14 ^b^	N.D.	0.19 ± 0.00 ^a^	631.03
‘Gihyun gold no. 3’	620.87 ± 2.45 ^a^	36.54 ± 2.06 ^e^	17.15 ± 0.13 ^a^	N.D.	0.11 ± 0.00 ^c^	674.67
‘Gihyun green’	381.59 ± 1.03 ^d^	92.64 ± 0.71 ^d^	7.01 ± 0.00 ^c^	N.D.	0.07 ± 0.00 ^d^	481.31
‘Gihyun red’	413.90 ± 1.78 ^c^	141.48 ± 0.75 ^c^	2.14 ± 0.02 ^e^	N.D.	0.07 ± 0.00 ^e^	557.59
‘Redlee’	345.93 ± 0.09 ^e^	162.42 ± 0.68 ^b^	6.17 ± 0.01 ^d^	3.46 ± 0.05	0.17 ± 0.00 ^b^	518.15

Results are the mean values ± SD from three technical measurements of ten biological fruits; means in the same column with superscript with different letters (a, b, c, d, and e) are significantly different at *p* < 0.05. N.D., Not detected.

**Table 4:** Individual sugar contents (g/100 g FW) of guava (*Psidium guajava* L.) fruit.

Cultivar	Fructose	Glucose	Sucrose	Total Sum
‘Gihyun gold no. 2’	4.46 ± 0.02 ^b^	3.48 ± 0.03 ^a^	0.41 ± 0.03 ^d^	8.35
‘Gihyun gold no. 3’	2.53 ± 0.05 ^c^	1.33 ± 0.01 ^c^	2.44 ± 0.06 ^b^	6.30
‘Gihyun green’	4.35 ± 0.19 ^b^	2.58 ± 0.09 ^b^	0.15 ± 0.01 ^e^	7.08
‘Gihyun red’	5.41 ± 0.16 ^a^	3.44 ± 0.10 ^a^	1.35 ± 0.12 ^c^	10.20
‘Redlee’	2.35 ± 0.01 ^c^	1.43 ± 0.04 ^c^	4.84 ± 0.05 ^a^	8.62

Results are the mean values ± SD from three technical measurements of ten biological fruits; means in the same column with superscript with different letters (a, b, c, d, and e) are significantly different at *p* < 0.05.

**Table 5:** Polyphenol contents (mg/100 g FW) of guava (*Psidium guajava* L.) fruit.

Cultivar	(+)-Catechin Hydrate	Rutin Hydrate	Epigallocatechin Gallate	Ferulic Acid	Gallic Acid	Epicatechin	Rosmarinic Acid
‘Gihyun gold no. 2’	10.07 ± 0.18 ^d^	11.34 ± 0.07 ^c^	0.81 ± 0.02 ^c^	0.95 ± 0.01 ^a^	0.38 ± 0.01 ^c^	0.53 ± 0.02 ^a^	0.07 ± 0.00 ^b^
‘Gihyun gold no. 3’	55.55 ± 0.53 ^a^	15.93 ± 0.12 ^b^	7.48 ± 0.33 ^a^	0.09 ± 0.00 ^e^	0.44 ± 0.01 ^a^	0.39 ± 0.03 ^b^	0.12 ± 0.00 ^a^
‘Gihyun green’	10.07 ± 0.15 ^d^	3.62 ± 0.01 ^e^	0.34 ± 0.01 ^d^	0.59 ± 0.01 ^b^	0.40 ± 0.02 ^b^	N.D.	N.D.
‘Gihyun red’	19.66 ± 0.05 ^c^	6.13 ± 0.05 ^d^	1.06 ± 0.02 ^c^	0.50 ± 0.01 ^c^	0.21 ± 0.01 ^e^	N.D.	N.D.
‘Redlee’	36.81 ± 1.15 ^b^	64.81 ± 0.73 ^a^	3.69 ± 0.06 ^b^	0.36 ± 0.01 ^d^	0.34 ± 0.01 ^d^	N.D.	N.D.

Results are the mean values ± SD from three technical measurements of ten biological fruits; means in the same column with superscript with different letters (a, b, c, d, and e) are significantly different at *p* < 0.05. N.D., Not detected.

**Table 6:** Lycopene and total ascorbic acid contents (mg/100 g FW) of guava (*Psidium guajava* L.) fruit.

Cultivar	Lycopene	Total Ascorbic Acid
‘Gihyun gold no. 2’	5.21 ± 0.20 ^a^	114.43 ± 1.48 ^e^
‘Gihyun gold no. 3’	N.D.	198.27 ± 3.91 ^c^
‘Gihyun green’	N.D.	214.50 ± 6.10 ^b^
‘Gihyun red’	N.D.	164.96 ± 4.27 ^d^
‘Redlee’	3.60 ± 0.77 ^b^	292.38 ± 4.40 ^a^

Results are the mean values ± SD from three technical measurements of ten biological fruits; means in the same column with superscript with different letters (a, b, c, d, and e) are significantly different at *p* < 0.05. N.D., Not detected.

## Data Availability

The original contributions presented in this study are included in the article. Further inquiries can be directed to the corresponding author.
